# Simultaneous duodenal and ileocolic obstruction in Crohn’s disease: a case report

**DOI:** 10.1093/jscr/rjag063

**Published:** 2026-02-12

**Authors:** Hadeel Bin Shuiel, Alanoud Alsamari, Yaser AlShayaa

**Affiliations:** College of Medicine, AlFaisal University, Takhassusi Street, Al-Maather District, Riyadh 11533, Saudi Arabia; General and Colorectal Surgeon, Department of Surgical Oncology, King Fahad Medical City, Prince Abdulaziz Ibn Musaid Ibn Jalawi St, As Sulimaniyah District, Riyadh 12231, Saudi Arabia; General and Colorectal Surgeon, Department of Surgical Oncology, King Fahad Medical City, Prince Abdulaziz Ibn Musaid Ibn Jalawi St, As Sulimaniyah District, Riyadh 12231, Saudi Arabia

**Keywords:** Crohn’s disease, duodenal obstruction, ileocolic stricture, duodenojejunostomy, infliximab

## Abstract

Duodenal involvement in Crohn’s disease (CD) is a rare phenomenon, often presenting with significant diagnostic and therapeutic challenges. We report the case of a male patient in his early 50s with longstanding CD and prior right hemicolectomy who presented with subacute obstructive symptoms, severe weight loss, and failure of medical therapy including corticosteroids and biologics. Imaging revealed strictures in both the duodenum and the ileocolic anastomosis site. Surgical intervention involved resection of the strictured duodenal segment with duodenojejunostomy and ileocolic resection with primary anastomosis. Postoperative recovery was uneventful, and the patient remained symptom-free during follow-up on infliximab therapy. This case highlights the importance of timely surgical intervention in complex CD and supports a multidisciplinary management approach.

## Introduction

Crohn’s disease (CD) is a chronic, relapsing inflammatory bowel disease that may affect any part of the gastrointestinal tract. While terminal ileum and colon involvement are most common, upper gastrointestinal tract manifestations, including duodenal disease, occur in approximately 0.5%–4% of patients [1]. Duodenal CD (DCD) poses distinct clinical challenges due to its rarity, nonspecific symptoms, and potential for complications such as strictures, fistulas, and perforation [2].

Management strategies for DCD range from medical therapy, including corticosteroids, immunosuppressants, and biologics—to surgical intervention when complications like obstruction or perforation arise [3, 4]. With the increasing availability of biologic agents such as ustekinumab and infliximab, there is ongoing debate about the timing and necessity of surgical intervention in severe cases [4, 5].

We present a case of simultaneous duodenal and ileocolic obstruction in a patient with CD, successfully managed through surgical resection after failure of medical therapy. This case contributes to the limited but growing body of literature on complex presentations of DCD requiring operative management.

## Case presentation

A man in his early 50s with a 20-year history of CD presented with subacute vomiting, severe weight loss (35.2 kg, BMI 12.47), and progressive abdominal pain. His surgical history included a remote right hemicolectomy for bowel obstruction, with no subsequent follow-up. He denied diarrhea, rectal bleeding, fever, or extra-intestinal manifestations.

Initial laboratory findings revealed: WBC 4.5 × 10^3^/μL, hemoglobin 11.2 g/dL, platelets 316 × 10^3^/μL, CRP 8.5 mg/L, albumin 25.9 g/L, and creatinine 59 μmol/L. The patient had previously received prednisolone, Mesalamine, and a 10-month trial of adalimumab, without significant symptom resolution. He had recently received one dose of ustekinumab before admission.

**Figure 1:**
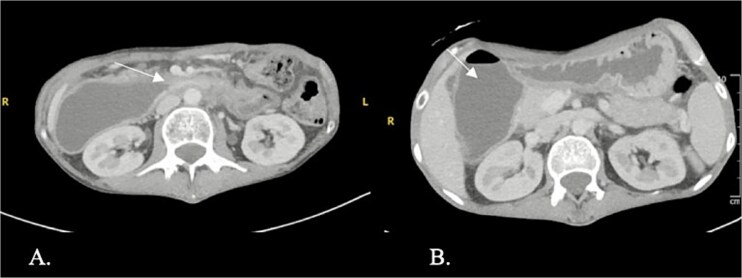
Axial contrast-enhanced computed tomography (CT) showing a stricture with thickening in the third and fourth parts of the duodenum (arrow) and proximal duodenal and gastric dilatation.

**Figure 2:**
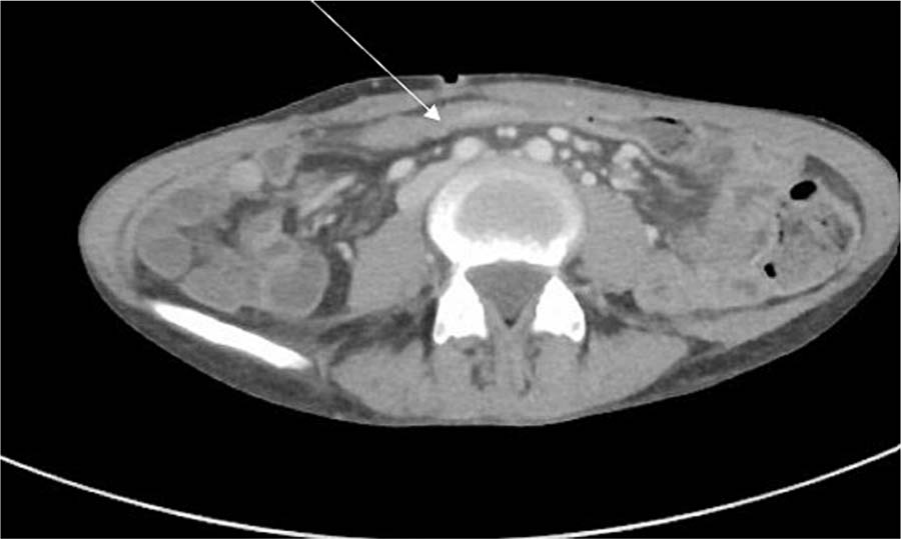
CT scan demonstrating thickening and abnormal enhancement of the neoterminal ileum (arrow), consistent with active CD.

**Figure 3:**
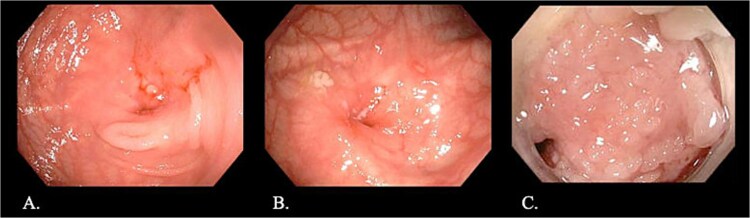
Endoscopic images: (A, B) Stricture at the ileocolic anastomosis with severely inflamed mucosa (arrows). (C) Non-traversable duodenal stricture observed during push enteroscopy.

CT enterography demonstrated strictures involving the third and fourth portions of the duodenum with proximal gastric dilatation (Fig. 1), inflammatory changes in the transverse colon mesentery, and thickening of the neoterminal ileum (Fig. 2). Push enteroscopy confirmed a non-traversable stricture with multiple ulcers in the third portion of the duodenum; biopsies indicated chronic duodenitis. Colonoscopy revealed a non-traversable stricture at the ileocolic anastomosis, with biopsies showing severely active chronic enteritis (Fig. 3).

After nutritional optimization with total parenteral nutrition (TPN) and withholding corticosteroids for two weeks, the patient underwent exploratory laparotomy. Dense adhesions were lysed. A stricture in the third and fourth portions of the duodenum was resected, followed by a stapled side-to-side duodenojejunostomy. A second stricture at the ileocolic anastomosis was similarly resected and reconstructed. Hemostasis was achieved and two drains were placed. The patient was extubated postoperatively in stable condition.

The patient remained NPO and on TPN during early postoperative recovery due to preoperative malnutrition. He developed postoperative pneumonia, which was successfully treated with piperacillin-tazobactam and vancomycin. Oral intake was resumed on postoperative day six, and he was discharged on Day 13. Histopathology confirmed CD in both resected segments without dysplasia.

Four weeks postoperatively, infliximab therapy was initiated. At one-year follow-up, he reported no gastrointestinal symptoms, with normalization of inflammatory markers and endoscopy showing healthy mucosa at both anastomotic sites.

## Discussion

Duodenal involvement in CD is uncommon but potentially severe [6]. It may manifest as strictures or ulceration leading to gastric outlet obstruction and frequently coexists with disease in other gastrointestinal locations [7]. The upper GI location makes endoscopic access and intervention more difficult, often delaying diagnosis and management [8].

Managing CD in the duodenum especially when the first or second part is involved can be particularly difficult. These areas lie close to key structures like the pancreas, bile duct, and major blood vessels, making surgical access and intervention more complex and riskier [9]. Strictures in this region are also less responsive to endoscopic treatment, and resection carries a higher chance of complications [9]. As Yamamoto *et al*. (2020) note, there is no universally accepted surgical approach for proximal duodenal Crohn’s, so management often needs to be tailored to each individual case [9]. While bypass procedures such as gastrojejunostomy are sometimes used, they come with potential drawbacks, including bile reflux and ulcer recurrence. For shorter or fibrotic strictures, stricturoplasty is often preferred, as it preserves bowel length and function [10]. The choice of techniques such as Heineke Mikulicz for shorter segments or Finney for longer ones depends on the stricture’s size and location [10]. Recent findings by Yang *et al*. (2023) suggest that stricturoplasty offers long-term outcomes like resection, but with a lower risk of postoperative complications such as: short bowel syndrome [10]. Ultimately, the choice of surgical strategies should reflect not just the stricture itself, but also the patient’s overall disease severity and surgical history [10].

In this case, despite trials of corticosteroids, mesalamine, and anti-TNF agents, the patient developed simultaneous obstructive lesions in the duodenum and terminal ileum, necessitating surgery. This supports prior findings that up to 50% of CD patients will eventually require surgical intervention despite advances in medical therapy [11].

A study clearly demonstrated that while bypass procedures such as gastrojejunostomy are frequently used for duodenal CD, they carry risks of recurrent ulceration unless combined with vagotomy [12]. However, more recent data suggest that proton pump inhibitors may effectively prevent recurrence, making vagotomy unnecessary [13].

Our patient had favorable outcomes with a resection-based approach and post-operative biologic therapy. This aligns with the growing evidence that multimodal therapy, combining surgery, nutritional support, and biologics, leads to better long-term control of severe CD [14, 15].

## Conclusion

Simultaneous duodenal and ileocolic obstruction in CD is rare but requires a high index of suspicion, especially in patients with failed medical therapy and obstructive symptoms. Early diagnosis, multidisciplinary evaluation, and timely surgical intervention are crucial to preventing severe complications. Postoperative biologic therapy may help maintain remission and improve long-term outcomes.
